# Correction: Analysis of internet of things implementation barriers in the cold supply chain: An integrated ISM-MICMAC and DEMATEL approach

**DOI:** 10.1371/journal.pone.0321608

**Published:** 2025-04-04

**Authors:** The PLOS One Staff

## Notice of Republication

This article was republished on March 10, 2025, to correct an error in the third author’s name. The correct name is: Md Abrar Jahin. The correct citation is: Ahmad K, Islam MS, Jahin MA, Mridha MF (2024) Analysis of Internet of things implementation barriers in the cold supply chain: An integrated ISM-MICMAC and DEMATEL approach. PLoS ONE 19(7): e0304118. https://doi.org/10.1371/journal.pone.0304118.

The publisher apologizes for the error. Please download this article again to view the correct version. The originally published, uncorrected article and the republished, corrected articles are provided here for reference.

## Supporting information

S1 File Originally published, uncorrected article. (PDF)

S2 File Republished, corrected article. (PDF)
